# Multiple objective energy optimization of a trade center building based on genetic algorithm using ecological materials

**DOI:** 10.1038/s41598-024-58515-8

**Published:** 2024-04-23

**Authors:** Elham Kabiri, Negin Maftouni

**Affiliations:** Department of Mechanical Engineering, Faculty of Engineering, Alzahra University, Tehran, Iran

**Keywords:** Building energy optimization, NSGA-II, Radiant ceiling cooling, Environmentally-friendly materials, Environmental sciences, Energy science and technology, Engineering

## Abstract

It is crucial to optimize energy consumption in buildings while considering thermal comfort. The first step here involved an EnergyPlus simulation on a trade center building located in Tehran, Bandar Abbas, and Tabriz, Iran. A multi-objective optimization was then performed based on non-dominated sorting genetic algorithm II (NSGA-II) in jEPlus + EA to establish the building in the selected city where would benefit the most from implementing the radiant ceiling cooling system. Efforts were undertaken to choose environmentally-friendly materials. The final solution by Pareto charts resulted in a 52% reduction in energy consumption, a 37.3% decrease in cooling load, and a 17.4% improvement in comfort hours compared to the original design. Annual emission of greenhouse gas reduced as 167.67 tone of CO_2_ equivalent emission, 25.77 ton of CH_4_, and 0.2 ton of NO_2_. The mentioned algorithm was conducted for the first time on a trade center, including a DOAS system and radiant ceiling cooling system. Simultaneously, the environmental-friendly materials were dealt with. The procedure holds significant relevance for the design and optimization of buildings in Iran, especially wherever the climate is hot and humid. This approach offers advantages to the environment by reducing the impact on energy resources and utilizing environmentally-friendly materials.

## Introduction

Supplying energy plays a crucial role in human life and progress. Studies indicate that buildings currently account for 32% of the world’s total energy consumption, a significant increase in recent years^1–4^. In Iran, over 40% of the total energy is consumed in commercial and residential buildings^5,6^. A large body of literature has been devoted to lowering building energy consumption. Advances made in the last two decades constitute initial steps towards designing buildings with low energy consumption^7^. In accordance with significant endeavors to accomplish this objective, the EU has pledged to reduce greenhouse gas emissions by 40% from 1991 to 2030 in order to promote sustainable and secure development^8^. Efforts were additionally undertaken to accomplish nearly zero-energy buildings^9,10^ by enhancing energy efficiency and finding solutions to how to reduce building’s energy consumption in different regions and climates^11–13^. There is a significant desire to incorporate environmentally-friendly materials in order to decrease the greenhouse gas emissions embedded with the material requirements of buildings as well. Moreover, different technologies proposed to reduce buildings energy consumption including radiant cooling systems.

Research suggest that the utilization of radiant ceiling cooling systems leads to a significant energy saving, ranging from 30 to 50%, when compared to conventional cooling systems^14^. ISO 11855^15^ classified radiant systems as radiant ceiling panels, embedded surface systems, and thermally-activated building systems. The advantages of radiant cooling systems include supplying or extracting heat through directly transferring radiant heat between the human body and radiant surfaces and indirectly through convection^16^. In 1937, the problem of ceiling condensation was partially fixed in a store in Zurich, Switzerland, using fresh air and airflow on radiant cooling surfaces. In 2012, Miril et al. simulated radiant heating and cooling systems and recommended a panel surface temperature of about 17 °C to avoid undesirable condensation and achieve a 10% energy saving^17^. In 2000, Gatley^18^ implemented a dedicated outdoor air system (DOAS) to control condensation by adjusting the panel temperature above the dew point. Given the failure of the radiant system alone to provide cooling or heating for a system, Fabrizio et al. coupled this system with an all-air system to improve its cooling performance. The radiant system demonstrated a 5.5% increase in efficiency compared to the conventional all-air system, while the DOAS showed a 30% higher energy saving rate than the traditional radiant system^19^. Radiant cooling systems were discovered to decrease energy consumption by 34% in comparison to traditional variable air volume systems, while maintaining the same air conditioning level. Although radiant cooling systems have a higher initial cost compared to all-air systems, their payback period is approximately 2.5 years^20^. Moreover, these systems are economically viable for use in tropical regions. No research has been conducted on the analysis of how climate impacts the performance of these systems in the Middle East and Iran.

Besides various heating and cooling system types, another important parameter affecting the value of buildings about energy and environment issues is materials constructing different parts of buildings. Environmentally-friendly materials play a crucial role in designing advanced buildings. The husk of bamboo, coconut, and rice was thermally decomposed into biochar and added to concrete to improve its thermomechanical properties and decrease biowaste^21–23^. Marine clay has also been employed as a cement alternative to achieve sustainable concrete^23,24^. Bio-organic materials were utilized to manufacture PCMs^25,26^, while waste-derived fly ash was employed in the production of environmentally-friendly binders for the construction of sustainable buildings^27,28^. Moreover, making green bricks through geopolymerization and using slate tailings was also addressed in the literature^29^. In addition, significant effects of parameters such as wall layers, PCMs in various parts of buildings and different facades on buildings energy consumption were investigated in different dimensions^30–37^.

Design optimization constitutes an essential subject in building energy consumption. Complicated computational problems of decision-making models in this field can be solved using genetic algorithm method, which is based on simulating the genetic evolution of living organisms. This approach has been utilized in numerous research projects to discover optimal answers in optimizing buildings energy consumption and addressing the complexity of the issue. Sambou et al.^38^ employed genetic algorithms to optimize the thermophysical properties of multi-layer wall’s materials, aiming to improve comfort levels and reduce energy consumption in buildings. Indoor thermal capacitance and thermal resistance were objective functions. Hamdy et al.^39^ attempted to optimize the construction of a building to improve the energy efficiency and reduce the consequent greenhouse effect. Siddharth et al.^40^ developed an application to forecast the energy consumption of a building by implementing a database of the parameters generated through a genetic algorithm. Furthermore, Castelli et al.^41^ produced a model to predict the energy use of a building with a genetic algorithm. In 2016, Ramos Ruiz et al.^42^ considered the thermophysical properties of a building envelope and tried to optimize them to obtain maximum energy reduction. Ascione et al.^43^ utilized building automation systems with a genetic algorithm approach to enhance the energy saving of buildings. Hence, the genetic algorithm techniques with employing bio-inspired operators generate high-quality solutions for search and optimization problems, and therefore the study of natural processes has served as a basis for algorithms inspired by nature.

Among various heuristic methods, NSGA-II is widely used in building energy optimization problems. The strategy relies on the process of natural selection and the survival of the most adapted organisms and is remarkably unforeseeable and flexible^44^. For instance, Delgarm et al. introduced a novel approach to resolve simulation-based multi-objective optimization challenges while addressing the main constraints on building energy performance. They also integrated both single- and multi-objective optimization algorithms utilizing NSGA-II within EnergyPlus^45,46^. Ascione et al. minimized the total cost of energy, discomfort hours, and primary energy consumption (to 62.0–91.9 kWh/m^2^) in the conventional residential buildings of four regions in Italy using genetic algorithm in MATLAB/EnergyPlus^47^. Salata et al. successfully decreased the annual operating cost of energy and greenhouse gas emissions in 14 European cities through the implementation of a multi-objective optimization based on active NSGA-II^48^. Jahani et al. conducted a study utilizing a genetic algorithm-based numerical moment matching approach to minimize the electrical power usage of 8370 urban buildings. The study focused on three key variables: region, cooling efficiency, and attic insulation. The findings revealed a significant reduction in electricity consumption, estimating a decrease of 10.219 kWh for the buildings. This indicates a potential 6% saving in annual electricity consumption. This method can use a small sample of houses as a representative to show the energy consumption behavior of a group of buildings^49^. Naderi et al. employed NSGA-II to optimize the model of a typical office room on the middle floor of a building based on climate and window orientation in EnergyPlus. They simultaneously minimized the total annual building electricity consumption, predicted percentage of dissatisfied (PPD) and discomfort glare index as three objective functions. The obtained results suggested improvements in the thermal and visual comfort of the occupants and reductions in electricity consumption^50^.

Baghoolizadeh et al. conducted research on multi-objective optimization of Venetian blinds in office buildings. Energy simulations were conducted using EnergyPlus and jEPlus software, considering various design variables. Optimization using the NSGA-II algorithm were performed to extract optimal points on a Pareto front. The findings indicated notable reductions in energy consumption and enhancements in visual and thermal comfort, with optimal points identified based on different weight coefficients. The results of the study indicated that optimizing Venetian blinds in office buildings can lead to significant reductions in energy consumption while improving visual and thermal comfort for occupants. The optimization process involved sensitivity analysis and multi-objective optimization using the NSGA-II algorithm^51^. In this form of optimization, the consistency of the status of each objective function remains unchanged, leading to the acquisition of a non-dominant solution set. Consequently, this facilitates the derivation of simulation conclusions that are more applicable. In the forthcoming years, Pareto-based multi-objective optimization algorithms will emerge as the predominant approach for addressing optimization issues.

Chen et al. discussed the optimization and prediction of energy consumption, light, and thermal comfort in teaching building atriums using the NSGA-II algorithm and machine learning. The study addressed the challenges of balancing energy efficiency and indoor comfort in teaching buildings, particularly focusing on atrium design. The research involved field research, modeling, optimization, and evaluation of two atriums in a college teaching building in Jinan. By utilizing the NSGA-II algorithm and machine learning, the research aimed to enhance energy efficiency, light, and thermal comfort in atriums. The study demonstrated improvements in energy consumption and comfort levels through optimization strategies and predictive modeling. The results of the study revealed that by utilizing the NSGA-II algorithm and machine learning techniques during the optimization process, significant enhancements were achieved in energy efficiency, lighting, and thermal comfort in teaching building atriums. The optimization strategies led to reduced energy consumption, enhanced visual and thermal comfort, and overall improved performance of the atriums. The study identified optimal solutions and design factors that contributed to these improvements, showcasing the effectiveness of the approach in optimizing building performance^52^.

Numerous studies have been conducted on multi-objective energy optimization of buildings, as well as incorporating environmentally-friendly materials in building constructions. However, only a small number of studies address both aspects, with none of them examining the situation in Iran in a comprehensive research. There is a scarcity of research on trade center buildings’ energy optimization. Hence, in the current study, The NSGA-II optimization process carried out in jEPlus + EA focused on enhancing thermal comfort levels in the commercial center while minimizing energy consumption costs. Various building orientations, and diverse types of windows and shading, and different insulations and façades were contemplated. Besides, environmentally-friendly materials were considered along with other conventional materials. Additionally, for the first time, the effect of climate on the performance of radiant ceiling cooling systems was studied in Iran. The results showed a significant reduction in energy consumption through the implementation of this approach, thus making progress towards sustainability goals and lowering greenhouse gas production.

## Methodology

Given the complexity of designing buildings and the need for optimization before executing construction projects, different types of building energy simulation software have been developed for engineers in this field, e.g., EnergyPlus, TRNSYS, and DesignBuilder. In 2004, Americans introduced EnergyPlus as a potent simulation engine. This installation software functions based on climatic conditions and calculation of the heating and cooling loads of buildings^53^. Despite its numerous capabilities, EnergyPlus cannot be regarded as an optimization tool by itself. Therefore, it is necessary to utilize a second software package for this specific purpose. The present study used the Java-based jEPlus + EA as the optimization program. The output design parameters of EnergyPlus are used as objective functions in jEPlus. JEPlus conducts a parametric analysis on the input parameters provided by EnergyPlus and TRNSYS^54^. EnergyPlus was validated as a robust software package using analytical, comparative, experimental, and executive tests conducted in accordance with the building energy simulation test (BESTEST)^55^.

### Thermal comfort

Thermal comfort is an essential criterion for selecting a shopping center by purchasers, reflecting satisfaction with a thermal environment. In an optimized building, thermal comfort refers to the state of mind that expresses satisfaction with the thermal environment. It involves the balance between the body’s heat production and heat loss, as well as the subjective perception of comfort by the building occupants. Achieving thermal comfort in an optimized building means that the indoor environment is designed and managed to provide occupants with conditions conducive to their well-being and productivity. The main factors affecting thermal comfort include activity level, clothing insulation, mean radiant temperature, air temperature, relative humidity, and air speed^56^.

Predicted Mean Vote (PMV) can be calculated using Eq. (1):

1 $${\text{PMV}} = {\text{ f}}\left( {{\text{I}}_{{{\text{cl}}}} ,{\text{M}},{\text{ t}}_{{\text{a}}} ,{\text{ t}}_{{{\text{mrt}}}} ,{\text{ p}}_{{\text{a}}} ,{\text{ v}}} \right),$$

where I_cl_ as the insulation level of clothing and M as the metabolic rate are related to individual conditions and t_a_ as air temperature, t_mrt_ as mean radiant temperature, p_a_ as relative humidity, and v as air speed (m/s) represent environmental parameters. PMV is a function of six parameters, and thermal comfort is achieved when PMV lies between − 0.5 and + 0.5. As another parameter derived from Eq. (2), the Predicted Percentage of Dissatisfied (PPD) should be below 10% for comfort to be achieved^56–58^. The predicted percentage of dissatisfied (PPD) index reports the percentage of occupants feeling dissatisfied by the thermal conditions. Discomfort Hours of shoppers in each shop was recorded while PPD exceeded 10%.

2 $${\text{PPD }} = { 1}00 - {\text{95exp}}\left( { - 0.0{\text{3353PMV}}^{{4}} - 0.{\text{2179PMV}}^{{2}} } \right).$$

### Environmentally-friendly materials

Modern environmentally-friendly materials have gained popularity in many countries for applications ranging from the foundation to facade of buildings. Moreover, concrete is a commonly-used material in Iran and its environmentally-friendly type has been successfully manufactured in this country. Khalil et al. investigated the production state of autoclaved aerated concrete and potential application of industrial waste to making environmentally-friendly concrete^59^. Wood is another environmentally-friendly material that can be used as a facade to add to the aesthetic dimension of buildings and reduce energy consumption. Using bio-sourced organic phase change material (Bio-PCM) as an environmentally-friendly insulation in a building can also significantly reduce energy consumption and keep the indoor temperature at comfortable levels^60^. Bio-PCMs absorb energy by changing their phase during the heating process and transfer energy to the environment during the next phase change of the cooling process^61^. Therefore, it has the capability to store and release substantial quantities of energy as either heat or cold when undergoing a phase transition. The utilization of this technology in diverse applications, such as building materials or thermal energy storage systems, strives to improve the efficiency of heating and cooling processes, thereby diminishing energy consumption and greenhouse gas emissions. The use of light insulators embedded with Bio-PCM particles has shown promise in smoothing and reducing thermal peak loads. Proper installation of energy-efficient insulation materials can minimize heat loss or gain, leading to reduced energy costs and lower greenhouse gas production^62,63^. Sajadi et al. found the potential of the southern strip of Iran with a high average daily solar insolation and humid climate to be the greatest compared to other regions in this country for embedding PCMs in building envelopes and therefore reducing demand for energy^64^. A good environmentally-friendly candidate, instead of traditional concrete, is autoclaved aerated concrete that benefits from light weight and recyclability^65^. With the rising energy demand, environmental concerns are becoming more prominent, with carbon dioxide being recognized as a harmful substance to human health. However, there is shortage of awareness of the environmental impacts of buildings in Iran. In this study, some environmentally-friendly materials were considered options among some traditional materials. The specific criteria thought about while proposing them were resulting in less building energy consumption, lower greenhouse gas production, and recyclability.

## Modelling

### Model specifications

**Figure 1:**
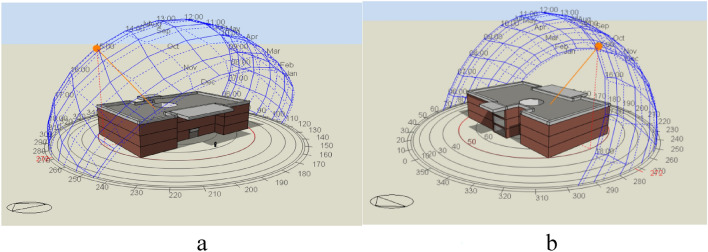
A 3D model of the building: (**a**) north view, (**b**) south view.

The present article optimized materials and orientation and the ratio of overhand fine to the side margins of shading in a large trade center. Figure 1 shows a 3D model of the study building from the north and south.

The study method chosen was simulation, with the studied building encompassing a total area of approximately 3000 m^2^ and three floors, each with ten stores. Table 1 presents the building specifications, including its surface area, window type, temperature set points, and number of floors.

**Table 1:** Building parameters of the trade center.

Building type	Commercial
Floor area (m^2^)	3000
Number of floors	3
Window	20-mm multilayer glass
Air-conditioning	Electrical
Cooling temperature set-point (°C)	22
	Set back (°C) 18
	Cooling (°C) 22
	Set back (°C) 24

Table 2 presents the materials used in the walls and their specifications and properties for the base state. Table 3 summarizes the U values ​of the building walls based on Iranian National Building Code.

**Table 2:** Properties of the materials used in the base state^65^.

Material	Conductivity (W/m °K)	Specified heat value (J/kg °K)	Density (kg/m^3^)
Brick	1	840	1900
Concrete	0.34	840	1300
Bitumen	0.25	1000	1700
Mortar	1.15	920	2000
Bitumen and felt	0.5	1000	1700
Granit	2.9	840	2500
Ceramic	1.3	840	2300
Asphalt	1.15	840	2330
Gypsum	0.7	1000	1300
Sand	0.52	180	2050
Ceramic	1.3	840	2300

**Table 3:** Specifications of the building materials^66^.

Element	Property	Value (W/m^2^ **°**K)
Interior wall	Total heat transfer coefficient (U)	2.58
Exterior wall	Total heat transfer coefficient (U)	0.7
Floor	Total heat transfer coefficient (U)	1.45
Ceiling	Total heat transfer coefficient (U)	1.68

The rooms were equipped with a DOAS as the ceiling cooling air conditioner, whose capacity was automatically calculated based on the hottest days of the year. By default, the building was considered North oriented with a 0° angle, and the summer comfort temperature was set at 22 °C during the working hours of the shopping center. The activity level of the shoppers was considered 125 W/m^2^ and their type of summer clothing calculated based on ASHRAE 55. The conditions of other spaces such as stairways, halls, and facilities were assumed at standard levels. Fixed double-glazed windows with a thickness of 20 mm, height of 1.5 m, and a window-to-wall ratio of 30 were mounted on the middle of the outer wall of the building corridor. The capacity of the ceiling cooling air conditioner in the stores was automatically calculated derived from the hottest days of the year. The activity level of the shoppers was determined to be 125 W/person, taking into account the surface area of each store. Additionally, their clothing level was dynamically obtained as skin-covering summer clothing based on ASHRAE 55^56^. The amount of fresh air entering the room was also assumed to be 9.44 L per second per person. The explanations provided for Tehran (hot and dry), Bandar Abbas (hot and humid), and Tabriz (cold mountainous) were examined. Table 4 presents the climatic characteristics of these cities.

**Table 4:** Main climatic characteristics of the study cities.

Site	Bandar Abbas	Tehran	Tabriz
Climate	Dwa	BSk	Cfb
Climate zone	1B	3B	4B
Latitude	27/22° N	35/68° N	38/08° N
Longitude	56/37° E	51/32° E	46/28° E
Elevation (m)	10	1191	1351
Avg. air temp. (°C)	22	22	22
Min. air temp. (°C)	18	18	18
Max. air temp. (°C)	26	26	26

### Climatic regions of Iran

This study examined and simulated a trade center in Bandar Abbas, Tehran, and Tabriz with three different climates. The results of using radiant ceiling cooling systems were compared with those of conventionally used direct expansion (DX) to determine the city with the minimum cooling load for the next optimization step. A genetic algorithm was utilized to conduct a multi-objective optimization aimed at minimizing the costs.

### Optimization method

The present study was conducted to save energy and maximize individuals’ comfort by optimizing several design parameters and employing three different strategies. The first strategy entailed choosing the most suitable location to explore the optimal state with special modifications, leading to the evaluation of RCP-based cooling systems combined with DOAS in various climates of Iran. The building load was studied in seven hot months in Tabriz with a cold and harsh mountainous climate, Tehran with a hot and dry weather, and Bandar Abbas with a hot and humid weather in two modes of the radiant ceiling cooling system and conventional DX. The ceiling radiant cooling system was investigated as the standard DX system without DOAS and the system with DOAS. The second strategy focused on identifying the materials, building orientation, and their optimal specifications. In order to accomplish this goal, one of evolutionary algorithms was implemented.

### Non-dominated sorting genetic algorithm II simulation (NSGA-II)

Evolutionary methods are commonly used as generally search-based algorithms in optimizing complex problems. Genetic Algorithm (GA) is a problem-solving technique that draws inspiration from genetics and biological evolution. It is widely applied across various industries to find the best possible solution. GA operates as a competitive process, where only the fittest individuals survive while the less adaptable ones are gradually eliminated. Those individuals that survive are considered to possess excellent genes, whereas those that are eliminated represent the filtered-out genes.

In GA, data is manipulated through combining individuals, referred to as chromosomes, together. The selection process involves choosing the best chromosomes from the population to progress to the next iteration, where they have the opportunity to reproduce as parents. Through crossover, new chromosomes with characteristics from the parent chromosomes can be generated. Mutation, which is a rare occurrence in genetics, involves selecting a smaller value during the optimization process. Overall, GA follows the principles of genetics and biological evolution to iteratively search for the optimal solution through selection, crossover, and mutation processes.

A limited population of possible solutions is used in the genetic algorithm as an evolutionary algorithm^67^. Genetic algorithm obviates the need for exploring the entire search space and testing all the possibilities, which is very time-consuming. Chantrelle et al.^68^. performed different multi-objective optimizations with cost, energy consumption, and thermal comfort constraints. Wang et al. designed a green building by optimizing costs and environmental effects and recommended evolutionary algorithms for searching in multi-objective optimization.

NSGA-II is capable of finding optimal solution(s) by testing only several potential solutions; nevertheless, the result can be counted on as that obtained by examining all of the possible answers.

Compared to other genetic algorithms, the multi-objective evolutionary algorithm of NSGA-II provides a higher convergence in Pareto and helps with a more extensive examination of solutions in EnergyPlus^69^. Equation (3) presents the multi-objective optimization problem^48^.

3 $${\text{min }}\{ {\text{f}}_{{1}} \left( {\text{x}} \right),{\text{ f}}_{{2}} \left( {\text{x}} \right) \cdots {\text{f}}_{{\text{k}}} \left( {\text{x}} \right)\} {\text{ x}} \in {\text{F}},$$

$${\text{R }} \to {\text{ R}}^{{\text{n}}} ,{\text{ for i }} = { 1}, \ldots ,{\text{ k}},$$

where R^n^ represents the space in which x is located and R^k^ is the objective space. Here k ≥ 2 is the number of objective functions, and f (x) ∈ R^n^ is their vector in which fi(x): R^n^ → R^1^.

The weighted sum method^70^ has been used to find the final optimal solution between the Pareto points.

4 $${f}_{ws} \left({\text{x}}\right)=\sum_{{\text{i}}=1}^{2}{a}_{{\text{i}}}\frac{{{\text{f}}}_{{\text{i}}}\left({\text{x}}\right)-{{{\text{f}}}_{{\text{i}}}\left({\text{x}}\right)}^{{\text{min}}}}{{{{\text{f}}}_{{\text{i}}}\left({\text{x}}\right)}^{{\text{max}}}-{{{\text{f}}}_{{\text{i}}}\left({\text{x}}\right)}^{{\text{min}}}},$$

5 $${\sum_{i=1}^{k}{a}_{i}=1, a}_{i}\in \left[0,1\right],$$

where f_i_(x) is either the annual electricity consumption or discomfort hours as an objective function, f_i_(x)^min^ and f_i_(x)^max^ the minimum and maximum values of the objective function, respectively, and a_i_ the weighting factor of the objective function. They are optimized independently.

*a*_2_ is obtained using Eq. (6).

6 $${{\text{a}}}_{2}=\frac{1-{{\text{a}}}_{1}}{2} .$$

The multi-objective optimization problem was therefore converted to a single-objective problem with *a*_1_ as its single decision parameter used in minimizing *f*_*ws*_. The NSGA-II parameters were set as follow: the population size was considered 10, the maximum number of generations 100, the crossover rate 90% and the mutation rate 20%.

To select materials for the optimal state, the continuous variable of building orientation and types of window and shading (in case of the ratio of overhang acceptable to the side margins) were used in several models as per Table 5, which also presents discrete variables such as insulation, window, concrete, and different conventional facades with other insulations such as PCM, extruded polystyrene and expanded polystyrene as well as aerated concrete, autoclaved aerated concrete and concrete block and traditional facades of marble, brick, and wood in building optimization. In addition, Table 6 presents the properties of the materials.

**Table 5:** Optimization models.

Optimal type	Model 1	Model 2	Model 3	Model 4	Model 5	Model 6
Shading overhang fine/side	0	0.25	0. 5	0. 75	1	–
Window	Clear	Absorption	Reflective	Abs-Ref	Lew-E	Spectral s
Orientation	0 until 360

**Table 6:** Materials specifications for optimization.

Optimal type	Material	Conductivity (w/m °K)	Specific heat (j/kg °K)	Density (kg/m^3^)
Insulation	Extruded polystyrene with CO_2_	0.034	1400	35
	Expanded polystyrene	0.035	1400	25
	bio-sourced organic phase change materials (Bio-PCM)	0.2	1970	235
	Paraffin C13–C24-based PCMs)	0.21	3140	900
Concrete	Aerated concrete	0.24	1000	750
	Autoclaved aerated concrete	0.16	840	500
	Concrete block	0.51	1000	1400
Façade	Brick	0.72	840	1920
	Wood	0.12	1380	510
	Marble	2.77	802	2600

A large body of literature has been devoted to promoting the application of insulations such as PCM, autoclaved aerated concrete, and wood in environmentally-friendly buildings.

These specifications were required for only starting the optimization process, and the initial model did not encompass these alterations. The objective functions included annual electricity cost (IRR) and discomfort hours (hour), calculated only during working hours. Calculations were performed according to the yearly mean of PPD, which was reasonably below 10 in all the solutions obtained. Given the potential counteracting effects of decision variables on other objective functions, solutions should be derived based on the weight of the objective function; for instance, changing the variables to reduce the total power consumption might cause discomfort. Using a workstation with 2.6 GHz Intel™ Core i7-6700HQ quad-core processor, each optimization run took about 2 h.

According to the third strategy, the radiant system with the optimal materials and building state was examined in the selected city of Bandar Abbas, and the energy cost was estimated for the building. Adopting this strategy yielded a significant reduction in the cost of the building energy consumption, and the obtained solution suggested a proper perspective for constructing the building in this climate.

## Results and discussion

The following section illustrates and elaborates on the outcomes of the three mentioned strategies. The implications of the findings for energy consumption, occupant comfort, and environmental sustainability are also discussed.

### The first strategy

**Figure 2:**
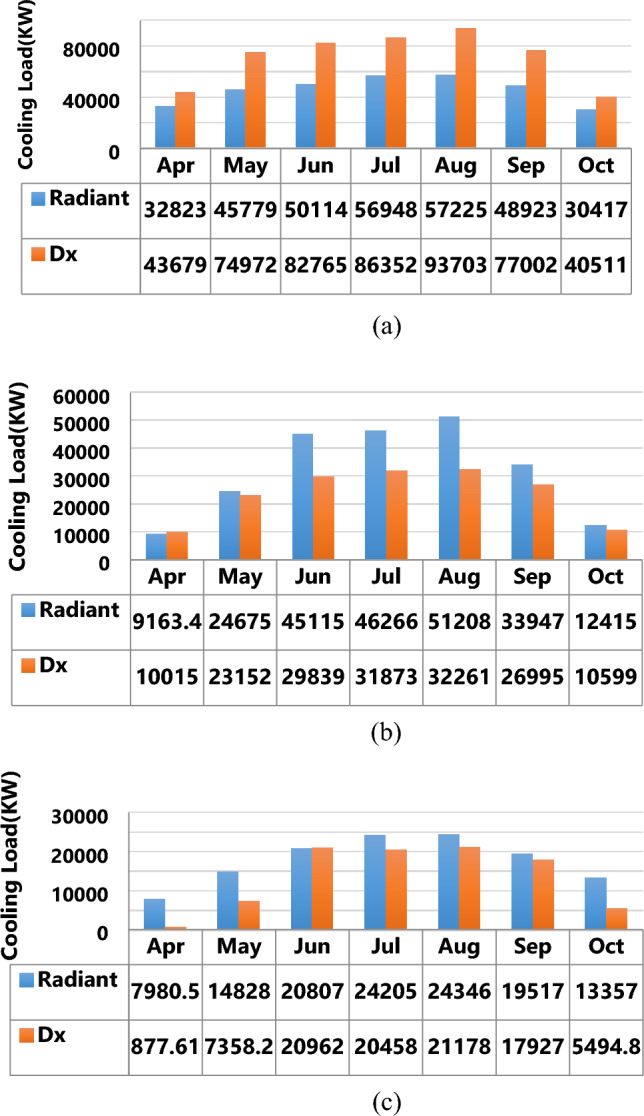
Reduced load of radiant cooling systems; (**a**) Bandar Abbas, (**b**) Tehran, (**c**) Tabriz.

According to the first strategy, which sought to identify an appropriate city for optimization, the conventional DX cooling system was compared with the radiant ceiling cooling system. Figure 2 illustrates the results of the cooling load comparison across the three cities.

The 34% reduction in the cooling load of the radiant system compared to that of the conventional DX system in Fig. 2a suggested the appropriateness of the radiant system for hot seasons in Bandar Abbas. The DX system was, however, recommended for Tehran and Tabriz in accordance with Fig. 2b,c. Bandar Abbas was more appropriate than Tabriz and Tehran for implementing the present project. Meaning that the most efficiency of energy resources was achieved there, and implementing radiant ceiling cooling system led to more energy saving in Bandar Abbas.

**Figure 3:**
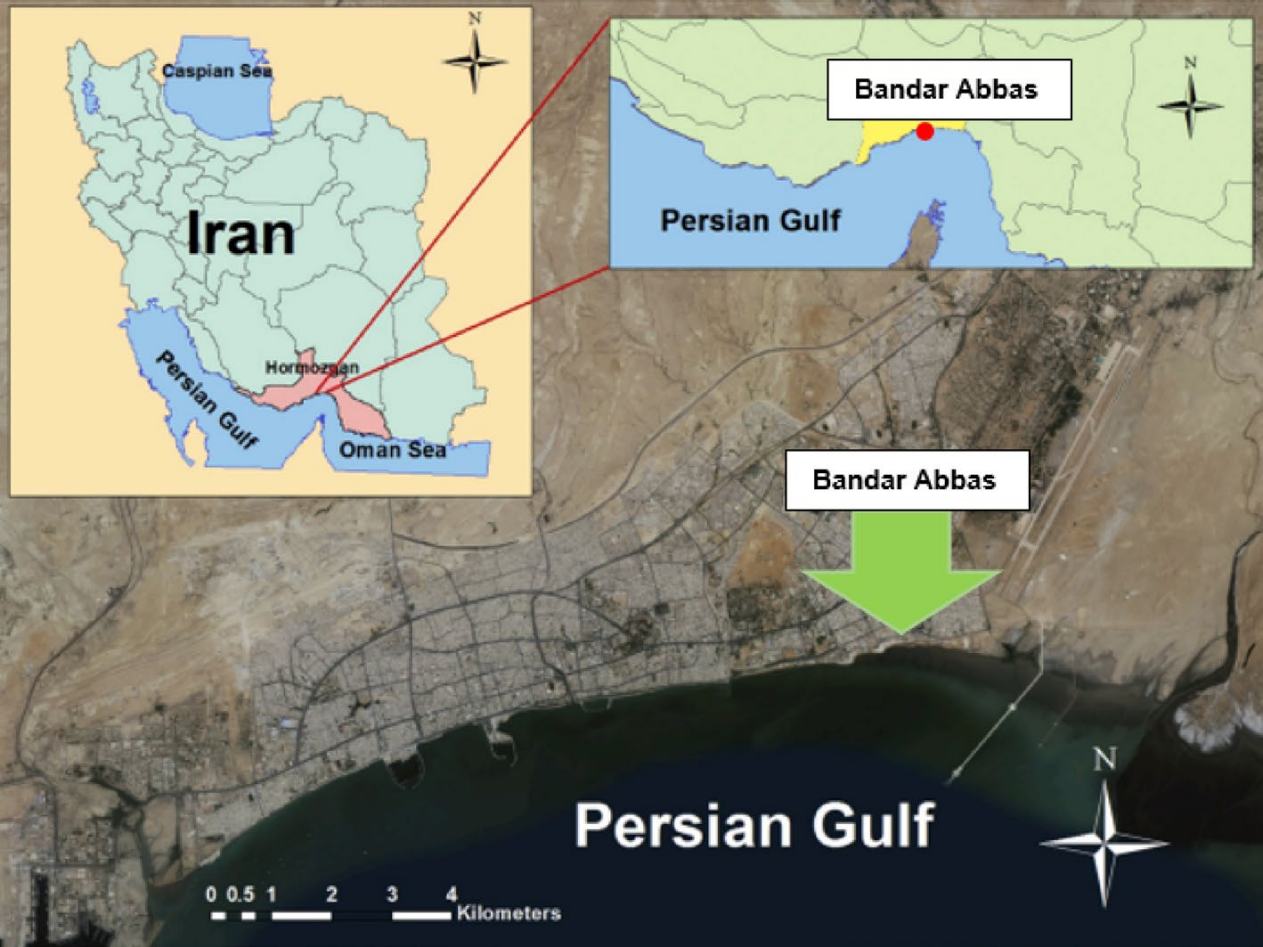
Geographical location of Iran and global climatic classification^71^.

Figure 3 shows the geographical location of Bandar Abbas in Iran, suggesting the appropriateness of the radiant ceiling cooling system given the hot and humid weather of this city in hot seasons. The DOAS was installed along with the radiant system to resolve the problem of ceiling condensation completely.

### The second strategy

**Figure 4:**
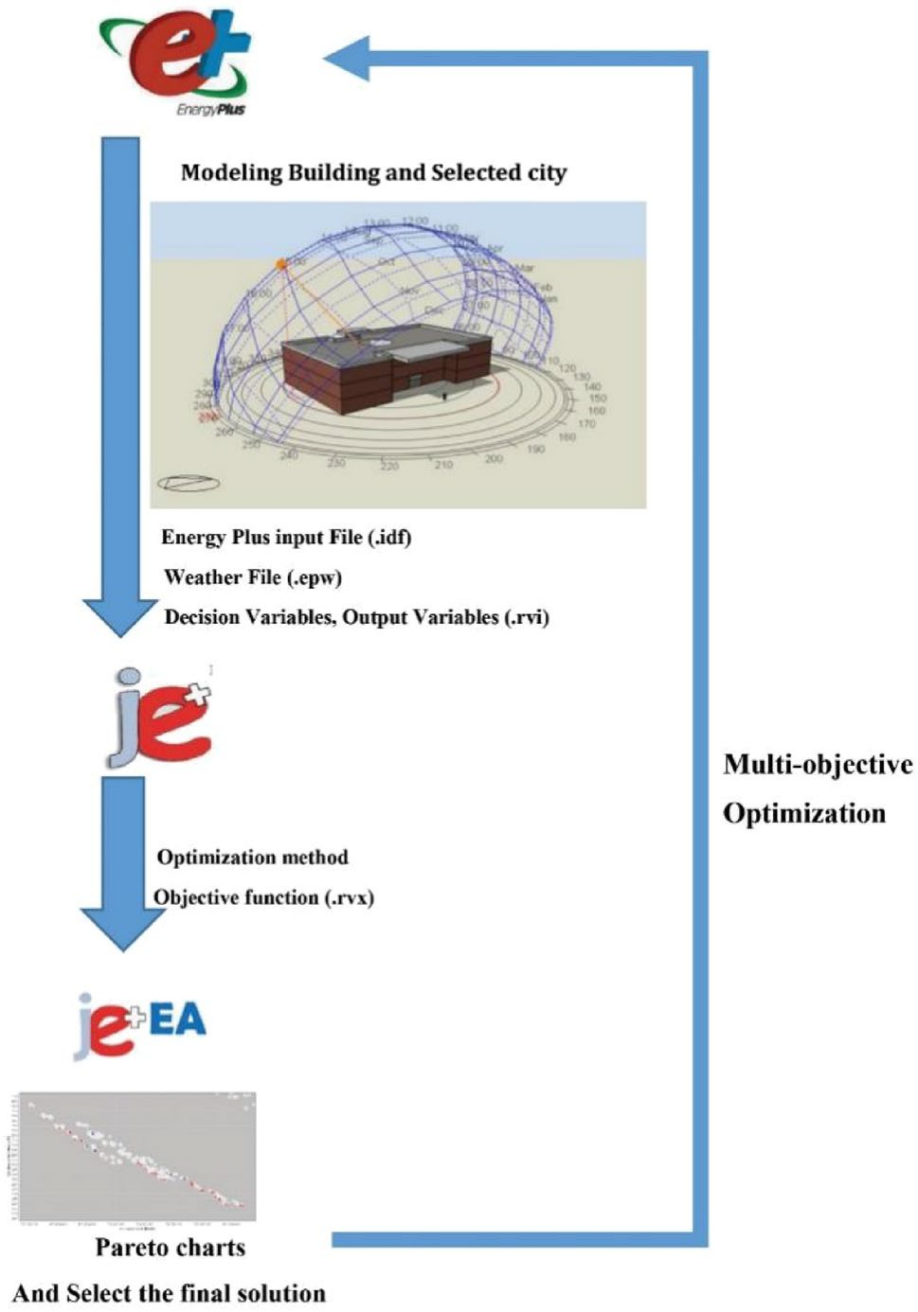
Building design in EnergyPlus and multi-objective optimization algorithm procedure.

Figure 4 illustrates a schematic diagram of the system based on the second strategy. After designing the building in EnergyPlus, the required files were extracted and entered into j plus. Optimization was then performed using NSGA-II. Optimal parameters selected from the Pareto chart were ultimately used in EnergyPlus to design the building.

**Figure 5:**
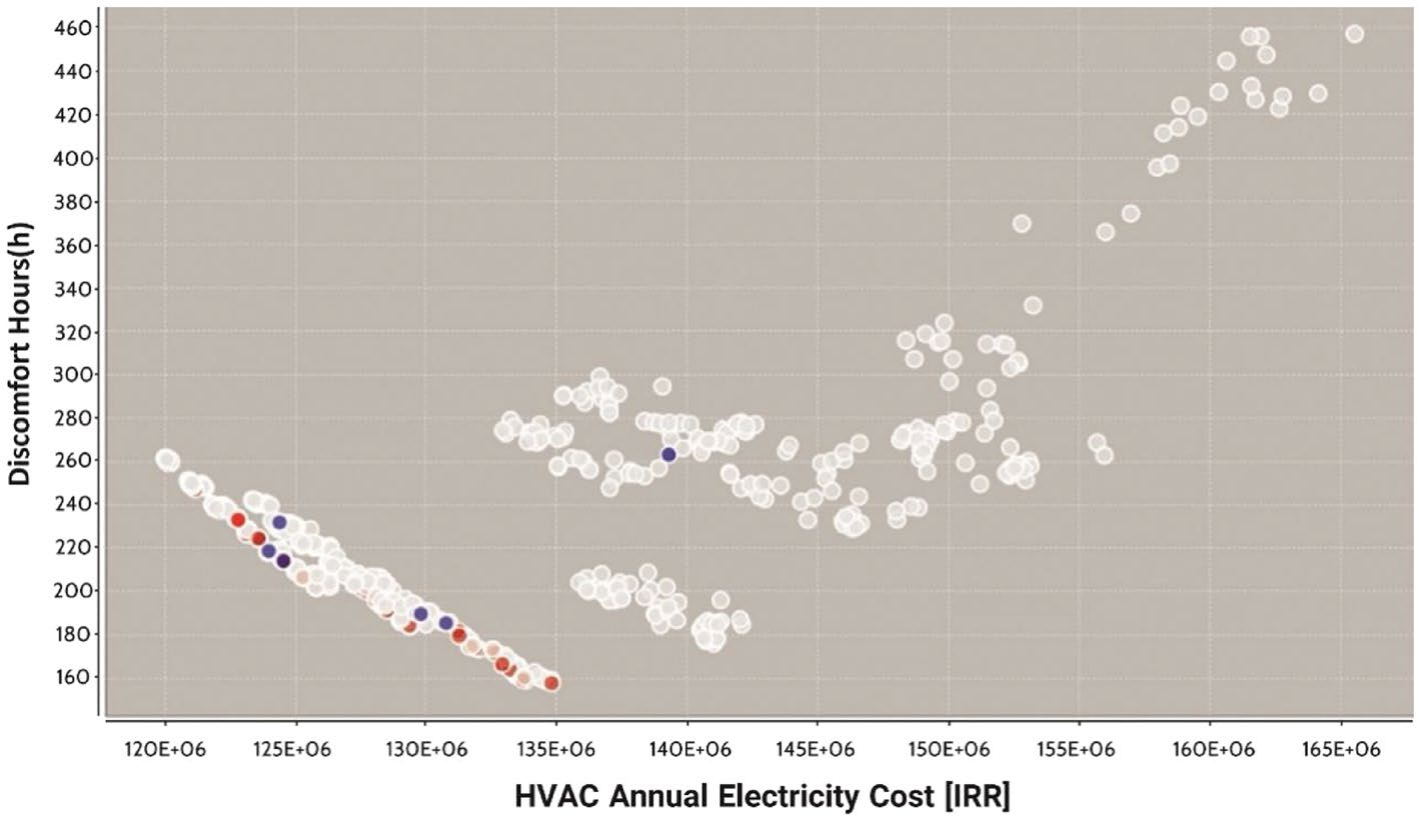
Identifying 129 optimal states by genetic algorithm in the entire population.

According to the second strategy, optimization could help decrease costs and integrate the construction with nature by paying more attention to natural resources and energy conservation in construction, particularly in hot and humid cities like Bandar Abbas. Moreover, the HVAC annual electricity cost, and discomfort hours objective functions are shown in Fig. 5 using Pareto charts of NSGA-II optimization. The Pareto graph typically illustrates the trade-offs between two or more objectives, with each point representing a potential solution and the set of non-dominated solutions forming the Pareto front. This visualization helps decision-makers in understanding the trade-offs among different objectives and choosing the most suitable solution according to their preferences. Overall, the Pareto NSGA-II graph serves as an essential tool for analyzing and visualizing the results of multi-objective optimization problems solved using the NSGA-II algorithm.

According to Fig. 5, analyzing 783 representative states for the entire study population in jEPlus + EA yielded 129 optimal states as the answers of this optimization problem after 100 generations. Table 7 shows the effect of *a*_1_ on the annual weight coefficient of power consumption, decision variables, and objective functions. The final solution was selected by focusing on energy consumption in the building. Efforts were made based on *a*_1_ in Table 7 to minimize both the annual power consumption and PPD as the index of residents’ comfort. The lower the energy consumption, the lower the cost.

**Table 7:** Effects of *a*_*1*_ on the annual electricity consumption weight, decision variables and objective functions in Bandar Abbas.

“a1” Coefficient	Slat angle (°)	Concrete	Insulation	Facade	Window	Shading	PPD (%)	Cost (lRR)	Annual electricity consumption (kWh)
0	360	Concrete block	Bio PCM	Brick	ECREF-1_COLORED_6MM	1	6.9	1.96E+08	894,023.4
0.1	45	Autoclaved aerated concrete (AAC)	Insul XPS	Brick	REF_A_CLEAR_LO_6MM	0.75	6.6	1.80E+08	88,039.58
0.2	180	Concrete block	Insul EPS	Wood	REF_A_CLEAR_LO_6MM	0. 5	7. 1	1.55E+08	86,278.39
0.3	0	Concrete block	PCM Wall	Brick	LoE_SPEC_SEL_CLEAR_6MM	1	6.51	1.53E+08	84,392.08
0.4	90	Concrete block	Bio PCM	Brick	LoE_SPEC_SEL_CLEAR_6MM	0.25	5.4	1.21E+08	83,892.08
0.5	180	Autoclaved aerated concrete (AAC)	Bio PCM	Wood	Reflectance	0. 5	5.6	1.26E+08	83,785.54
0.6	45	Autoclaved aerated concrete (AAC)	Insul EPS	Wood	REF_A_CLEAR_LO_6MM	0.25	6	1.20E+08	81,392.08
0.7	180	Concrete block	Bio PCM	Brick	LoE_SPEC_SEL_CLEAR_6MM	1	6.2	1.27E+08	74,392.08
0.8	90	Concrete block	Insul EPS	Marble	REF_A_CLEAR_LO_6MM	0.75	6.4	1.15E+08	70,392.08
0.9	0	Autoclaved aerated concrete (AAC)	Insul EPS	Brick	Reflectance	0.001	7.6	1.11E+08	68,432.08
1	180	Concrete block	Bio PCM	Wood	LoE_SPEC_SEL_CLEAR_6MM	0.25	7.8	1.07E+08	62,792.08

According to Table 7, residents’ comfort was ensured with a very low PPD even at a very high annual power consumption (*a*_1_ = 0, *a*_2_ = 0.5). It meant that the thermal comfort of occupants was warranted, and the indoor environment was designed and managed to provide occupants with situations that were suitable for their well-being. In addition to the comfort, reducing annual power consumption and costs is crucial. Given annual power consumption minimization as the main objective, strategies were adopted based on *a*_1_ to prevent solar heat from entering the room during the day and thus reduce the demand for power consumption in summer and increase comfort for residents. Using PCMs in walls and shadings, as well as new windows and wooden facades were very effective in this regard.

According to Table 7, increasing *a*_1_ reduces annual power consumption, costs, and comfort. In contrast, residents’ comfort rises at a low *a*_1_. As discussed earlier, energy consumption can generally decrease at the expense of thermal comfort reduction of residents. The vital mission of multi-objective optimization of building energy is to find the solution satisfying both thermal comfort and energy consumption criteria, while considering less impacts of the environment.

The current investigation solved the optimization problem utilizing *a*_1_ as a basis. It aimed to minimize power consumption and cost while ensuring the comfort of residents in a building located in Bandar Abbas. This was achieved by carefully selecting suitable decision variables, employing NSGA-II, and giving due consideration to the significance of environmentally-friendly materials. Using dehumidifiers based on PCMs and wood, shading, and reflective windows can help acclimatize to hot and humid conditions in Bandar Abbas. Moreover, *a*_1_ = 0.5 was employed in the formula.

**Figure 6:**
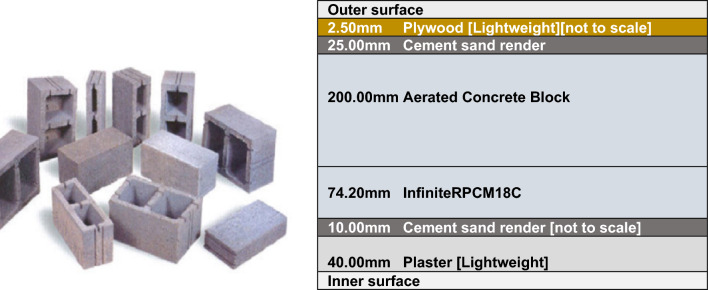
Materials selected from the optimization and used in the wall.

NSGA-II selects the most compatible states regarding cost reduction and environmental effects while ensuring the satisfactory performance and energy efficiency within the integrated building. According to these solutions, autoclaved aerated concrete and bio-PCM were respectively selected as lightweight environmentally-friendly concrete and insulation. Bio-PCM comprises materials derived from a combination of high-tech oils rather than conventional PCM technologies such as chemically-hazardous hexadecane. Bio-PCM is utterly innocuous for humans, animals, and the environment. It helps to improve the efficiency of cooling processes, thereby decreasing use of energy resources and greenhouse gas emissions. Autoclaved aerated concrete is a lightweight, cellular, and recyclable building material. It light weight leads to requiring less energy for cooling, hence less impact on energy resources, and producing less greenhouse gas and CO_2_ emission. Wood obtained as a facade from the solutions has a long history of application to integrating human structure and nature in facades. Figure 6 shows the materials identified in the optimization process and used in the wall.

The building orientation changing between 0° and 360° was optimized as 180° or southward to minimize the cost and maximize comfort. Moreover, the reflective type of window obtained from the optimization reduced the building load and energy consumption by reflecting the solar heat. In this answer, a shading ratio of 0.5 was implemented for shading analysis, which represents the ratio of overhang fine to the side margins of shading. However, it should be noted that with the importance of one of the objective functions (energy cost and comfort hours) the answer can change and other considered variables can be the answer to the problem. Figure 5 shows the optimal position of the radiant ceiling cooling system in Bandar Abbas compared to the no-change state among the 129 solutions, considering both cost and comfort of residents. In other words, no compromise was made among the two objective functions, i.e., cost and comfort.

### The third strategy

**Figure 7:**
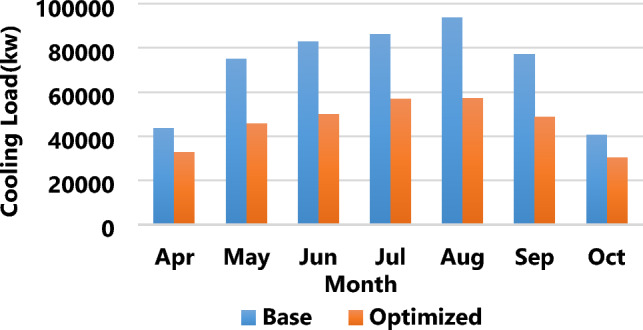
The optimized cooling load of the building versus the base load in Bandar Abbas.

**Figure 8:**
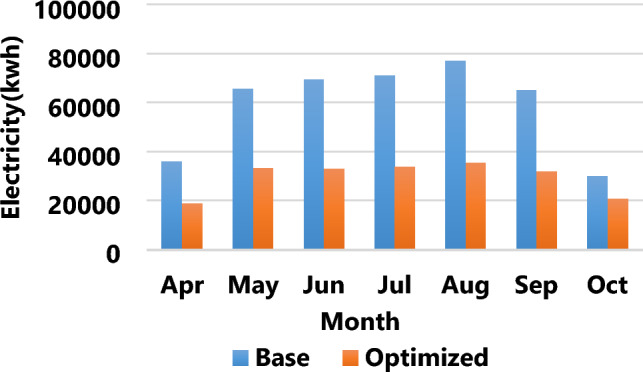
The optimized energy consumption of the building versus the base energy consumption in Bandar Abbas.

The results of the third strategy in Fig. 7 demonstrate an average building load reduction of 37.7% from April to October compared to the base state. Figure 8 shows a 52.3% reduction in energy consumption between the initial and optimized states in Bandar Abbas. A comparison of the results of multi-objective optimization showed that, considering the climate of Bandar Abbas, the total annual energy consumption of buildings amounted to 656,988.51 (kWh), experiencing a reduction of 52.3% to approximately 313,383.04 (kWh). This energy saving was equivalent to 663,305,205 IRR, and reduction of annual CO_2_ equivalent emission around 167.67 ton. Also, annual emission of CH_4_ decreased about 25.77 ton, and NO_2_ production, due to support energy needs of the building, lessened about 0.2 ton per a year. These vast amounts of greenhouse gas reductions play a crucial role in moving toward sustainable development and protecting the environment from damage.

The validation of EnergyPlus with analytical, comparative, experimental, and executive tests based on BESTEST ensures the reliability of the obtained results^58^. The results obtained using NSGA-II are also reliable, given its numerous applications in literature^45–49^.

### Analyzing the total cost of energy consumption

This study estimated the total cost of electricity consumption by the radiant ceiling cooling system in the optimal state and compared it with the cost of the all-air air system. The cost of electricity consumption is lower from March to November as the hot months of Bandar Abbas with hot and humid weather^72^.

Electrical energy was consumed by the radiant system for cooling the ceiling. In Iran, electricity is supplied and managed by Tavanir Organization, and according to its 2022 price list, the building cooling load costs 1500 IRR per kWh, and a progressive tax is applied to electrical energy^72^. After selecting the materials in the trade center of Bandar Abbas, the radiation system was compared with the situation after optimization. Given the unstable economic condition and highly volatile prices in Iran, the present study failed to perform an accurate financial analysis based on the material cost. Table 8 compares the energy saving in only one building floor between the pre- and post-optimization states.

**Table 8:** Electricity consumption on one building floor in hot months of the year.

Simulation status	Discomfort hours (h)	Electricity consumption (kWh)	Cost of electricity consumption (IRR)
Base with the radiant system (minimum solution)	247	95,119.86	142,679,790
Optimized (maximum solution)	158	81,219.86	121,829,710
Solution	204	83,785.54	125,678,319
Saving in percentage	17.4	0.12	0.12

### Limitations

One of the most arduous limitations faced by this project was the unstable economic climate in Iran, which impeded the ability to carry out precise and reliable economic computations and obtain accurate results. Furthermore, the limited availability of environmentally-friendly materials served as an additional constraint, narrowing down the choices that could be deemed suitable within the country.

### Implications for the future research

In upcoming studies, the impact of more valuable climates in Iran on the efficiency of radiant ceiling cooling systems could be explored. Additionally, the optimization of smart curtains and shadings to maximize energy conservation is recommended. Furthermore, investigating the ideal lighting conditions within the building is another intriguing aspect to be examined. Finally, Further research should be encouraged to investigate the impact of utilizing environmentally-friendly materials in order to address the environmental repercussions associated with the production of building materials, including waste generation and the release of greenhouse gas emissions. The original NSGA-II algorithm faced challenges with maintaining diversity and avoiding local optimum. Enhanced iterations of NSGA-II algorithm usually combines global search and greatly expand the range of solutions and leads to a more efficient handling of influential individuals, which should be duly acknowledged in the in forthcoming studies.

## Conclusion

This article proposed an environmentally-friendly model to reduce costs and achieve comfort for the occupants of a trade center using a ceiling cooling system. The project was designed in EnergyPlus, and the results were optimized based on NSGA-II in jEPlus + EA. The total annual electricity consumption of the building and indicators of individuals’ discomfort were used as objective functions. These criteria were employed to select the optimal state, control materials, and determine the orientation of the environmentally-friendly building.

- According to the first strategy, Bandar Abbas with a hot and humid climate was selected as the optimal city compared to Tabriz and Tehran for mounting the radiant ceiling cooling system.
- Installing DOAS along with the radiant system helped to resolve the problem of moisture and condensation.
- The results of the multi-objective optimization showed a 52.3% decrease in the total annual electricity consumption of the building and a reduction of 663,305,205 Iranian IRR in its cost in Bandar Abbas.
- Using Bio-PCMs and autoclaved aerated concrete as environmentally-friendly materials could help also to reduce the energy consumption of the building while supporting the thermal comfort of occupants.
- Less produced greenhouse gas, more recyclability, and slighter impacts on energy resources were achieved implementing environmentally-friendly materials.
- Quantities of annual emission of greenhouse gas reduction were: 167.67 tone of CO_2_ equivalent emission, 25.77 ton of CH_4_, and 0.2 ton of NO_2_.

The study’s research findings suggest several possible recommendations for practitioners and policymakers:

- This suggests that the optimization process led to significant improvements in both energy efficiency and cost savings for the building in Bandar Abbas.
- Encourage the adoption of radiant heating and cooling systems in commercial buildings, despite the high initial capital cost, as they can lead to long-term energy savings and improved occupant comfort.
- Provide public and private investors with information on the climate, energy efficiency, and cost effectiveness of using radiant ceiling cooling systems in commercial construction to encourage investment in these technologies.
- Emphasize the potential for significant energy savings by implementing solutions that comply with Iranian regulations and construction procedures, particularly in comparison to conventional local designs.
- Promote the utilization of environmentally-friendly materials in building construction to reduce electricity and energy consumption, as well as greenhouse gas emissions, while ensuring thermal and visual comfort for building occupants.

These recommendations aim to promote the adoption of energy-efficient and environmentally-friendly building practices in the commercial sector in Iran, as well as to support further research and exploration in this field.

## Data Availability

The datasets used and/or analyzed during the current study available from the corresponding author, Negin Maftouni on reasonable request.
